# Identification of Pyrrole-2-Carboxylic Acid from the Biocontrol Agent *Lysobacter* Involved in Interactions with Fusarial Fungi

**DOI:** 10.3390/microorganisms13061202

**Published:** 2025-05-24

**Authors:** Vishakha Jayasekera, Yong Han, Liangcheng Du

**Affiliations:** 1 Department of Chemistry, University of Nebraska-Lincoln, Lincoln, NE 68588, USA; udesilvajayasekera2@huskers.unl.edu; 2 Nebraska Center for Integrated Biomolecular Communication, University of Nebraska-Lincoln, Lincoln, NE 68588, USA; 3 Edison Biotechnology Institute, Ohio University, Athens, OH 45701, USA; hany@ohio.edu; 4 Department of Chemistry and Biochemistry, Ohio University, Athens, OH 45701, USA

**Keywords:** *Lysobacter*, biocontrol, pyrrole-2-carboxylic acid, signaling, biofilm

## Abstract

*Lysobacter*, a genus of Gram-negative bacteria, is known for producing antibiotic compounds, making it a promising biocontrol agent against crop pathogens. As part of the soil microbiome, *Lysobacter* species cooccur with a variety of microorganisms in the ecosystem. However, little is known about bioactive natural products involved in *Lysobacter*’s interactions with other organisms. This study investigated interactions between *Lysobacter* sp. 3655 and two economically important fungal pathogens, *Fusarium graminearum* and *Fusarium verticillioides*. We discovered a *Lysobacter* molecule that is dramatically suppressed when co-culturing with the fungi, and the structure of this molecule was determined to be pyrrole-2-carboxylic acid (P2C). Chitin, a primary component of fungal cell walls, also suppressed P2C production in *Lysobacter*. Exogenous P2C addition promoted formation of *Lysobacter* biofilms within a range of concentrations, suggesting its potential role as a signaling molecule. Previously reported result showed that the mutation of the global regulator Clp in *Lysobacter enzymogenes* led to drastic increase of biofilm formation. Intriguingly, while P2C increased the biofilm formation in the wildtype of *L. enzymogenes*, it reduced the biofilms in the Clp mutant. Together, these findings reveal P2C as a novel signaling molecule mediating the interaction between *Lysobacter* and surrounding fungal species, highlighting its role in *Lysobacter* adaptation in response to environmental conditions.

## 1. Introduction

*Lysobacter* species are well-known for their broad-spectrum antimicrobial activities and have gained significant attention as biocontrol agents against plant pathogenic fungi [1,2,3]. These bacteria exhibit remarkable versatility, producing various bioactive compounds that target a wide range of pathogens. Their ability to secrete hydrolytic enzymes enables them to degrade fungal cell walls, while their production of secondary metabolites, including polycyclic tetramate macrolactams (PoTeMs), phenazines, and cyclic lipopeptides, contributes to antimicrobial activity. Additionally, *Lysobacter* species possess contact-dependent killing mechanisms such as type IV and type VI secretions systems (T4SS and T6SS) [4,5,6]. Together, these properties have made *Lysobacter* promising candidates for sustainable agricultural applications.

*Fusarium* is a genus of filamentous fungi responsible for devastating plant diseases [7]. Species of the genus pose a significant threat to global agriculture by causing losses in staple crops. Moreover, these pathogens are known for their ability to produce mycotoxins, such as fumonisins and deoxynivalenol (DON), which not only reduce crop productivity but also pose serious health risks to humans and livestock when contaminated food and feed are consumed [8]. Among them, *Fusarium verticillioides* and *F. graminearum* are particularly notorious. *F. verticillioides* is a major pathogen of maize, producing fumonisins that contaminate grains and pose severe health risks to humans and animals [9,10]. Its systemic infection capability allows it to persist within plant tissues, making eradication challenging. On the other hand, *F. graminearum* is the primary causative agent of Fusarium head blight (FHB) in wheat and barley, leading to substantial yield reductions and contamination with DON, a potent mycotoxin that disrupts food safety and marketability [11,12]. The ability of these pathogens to survive in soil and plant residues, coupled with their resistance to conventional fungicides, exacerbates their impact on global food security.

Recent studies have demonstrated that *Lysobacter* strains can effectively suppress many pathogenic fungi [3,13]. For instance, *L. enzymogenes* have been shown to inhibit the *Fusarium* species [14,15,16,17]. This species produces heat-stable antifungal factor (HSAF), a PoTeM that exhibits potent activity against various plant pathogens such as *Fusarium graminearum* and *Pythium ultimum*. Evidence indicated that HSAF inhibits the oxysterol-binding protein FgORP1 in *F. graminearum*, leading to compromised cell membrane integrity and disruption of ergosterol biosynthesis [15]. It also produces potent cyclic lipodepsipeptides against Gram-positive bacterial pathogens [18]. Additionally, *L. enzymogenes* secretes chitinases, glucanases, and lytic polysaccharide monooxygenases (LPMO) that degrade fungal cell walls, impairing fungal growth and colonization [16,19,20,21]. Interestingly, *L. enzymogenes* were shown to utilize outer membrane vesicles (OMVs) as delivery systems for HSAF and lytic enzymes including chitinases, glucanases, and LPMO [16,19]. These findings highlight the diverse antimicrobial strategies employed by *Lysobacter* species and their potential as effective biocontrol agents.

We have been studying biosynthetic mechanisms for *Lysobacter* antibiotics and *Fusarium* mycotoxins [1,22]. However, little has been done in molecular mechanisms underlying *Lysobacter–Fusarium* interactions. Despite many bioactive compounds having been isolated from *Lysobacter* species, essentially no bioactive molecule has been identified as a result of *Lysobacter–Fusarium* interactions. In this study, we chose a non-HSAF producing strain, *Lysobacter* sp. 3655, to interact with two fungal species. Although *L. enzymogenes* is the best understood species in terms of mode of actions for biocontrol activities, its production of the strong antifungal HSAF and analogs would not be ideal for *Lysobacter–Fusarium* co-culture system devised in this study. *Lysobacter* sp. 3655 is a relatively underexplored strain, and few research has studied its mechanism of biocontrol actions. However, there have been reports on the production of the potent antimicrobial compounds lysocins and the siderophore lysochelin [23,24]. In this study, we identified a small molecule from *Lysobacter* sp. 3655 that is involved in interactions with fungi. Our data indicated that this molecule could modulate biofilm formation. Bacterial biofilms are characterized by structural communities of sessile cells encapsulated by self-produced extracellular polymeric substances. Many pathogenic bacteria develop biofilms that are essential to colonize and further infect the hosts. As part of the soil microbiome, *Lysobacter* species cooccur with a variety of microorganisms and are subject to stress conditions in the ecosystem. In *L. enzymogenes*, a previous study indicated that biofilm formation facilitated the colonization of this bacterium in fungal hyphae [25]. A deeper understanding of the molecules that are involved in *Lysobacter–Fusarium* interactions could open new ways to control fungal diseases.

## 2. Materials and Methods

### 2.1. Materials, Strains, and Growth Conditions

Pyrrole-2-carboxylic acid (P2C) (purity 99%) was purchased from Sigma Aldrich, St. Louis, MI, USA. Bacterial and fungal strains used in this study are given in Supplementary Table S1. Luria–Bertani (LB) broth and agar plates were used for the routine growth of *Lysobacter* sp. 3655 (DSM), OH11, and OH11 Δclp supplemented with kanamycin (Km, 100 µg/mL) at 30 °C. Potato dextrose agar (PDA) was used for the cultivation of the fungal strains, *Fusarium verticillioides* and *Fusarium graminearum*, and for the anti-fungal assay. *Bacillus subtilis* was used for the anti-bacterial activity assay. Martin medium (0.5% peptone, 0.1% KH_2_PO_4_, 0.05% MgSO_4_·7H_2_O, and 1% glucose) was used in the metabolite fermentation of *Lysobacter* sp. 3655 and the fusarial fungi. Tryptic soy broth (TSB) medium was purchased from Sigma Aldrich and was used in biofilm formation studies.

### 2.2. Lysobacter-Fungal Co-Culture

Each fungal strain was inoculated in 100 mL of Martin medium and allowed to grow for 72 h in the incubator at 230 rpm at 30 °C. The co-cultures were prepared by adding 4 mL of Martin medium into an 8 cm sterilized dialysis tubing (MWCO of 6000–8000 Da) and 40 µL of bacterial inoculate into it. This dialysis tubing was then placed in the flask containing the 3-day old fungus. For the preparation of bacterial controls, the dialysis tubing containing the bacterial inoculum was placed in a flask of 100 mL Martin medium without the fungal culture. For the preparation of fungal controls, the dialysis tubing containing no bacteria and only 4 mL of Martin medium was placed in the flask containing 3-day old fungus. The co-cultured flasks were incubated at 230 rpm at 30 °C for 48 h. The metabolites from the dialysis tubing and the flask were extracted to acidified butanol (BuOH/0.05% TFA) in a 1:1 ratio and analyzed using High Performance Liquid Chromatography (HPLC) as per the protocol given below.

### 2.3. HPLC Analysis of Metabolites

The crude products were dissolved in 200 µL of methanol and an aliquot of 50 µL was analyzed by HPLC (1220 LC, Agilent Technologies, Memphis, TN, USA) which was equipped with a reverse-phase COSMOSIL (Nakagyo-ku, Kyoto, Japan) C_18_ column (4.6 × 250 mm, 5 µm particle size) and a UV detector set at 220 nm. The solvent system was water/0.05% formic acid (solvent A) and acetonitrile/0.05% formic acid (solvent B) with a flow rate of 1.0 mL/min. The solvent program was as follows: 5–40% B from 0–10 min, 40–60% B from 10–14 min, 60–100% B from 14–21 min, and maintained till 24 min, then back to 5% at 26 min and maintained till 30 min.

### 2.4. Isolation and Structure Elucidation of Pyrrole-2-Carboxylic Acid

A scale-up fermentation (2 L) of *Lysobacter* sp. 3655 was carried out in Martin medium at 30 °C for 48 h. The culture was extracted using acidified ethyl acetate containing 0.01% acetic acid, and the organic layer was collected by filtration and dried. A crude extract yield of 0.6 g was obtained and was subjected to separation by MPLC (Medium Pressure Liquid Chromatography) with 30 g reverse phase C18 silica gel eluted by a gradient 5% to 50% methanol. This afforded 3 subfractions. Fraction 1 was subjected to purification by HPLC using a Phenomenex Kinetex PS C18 column (10 × 150 mm, 3.5 µm particle size; Phenomenex Inc., Torrance, CA, USA). An isocratic elution of 18% acetonitrile afforded a white color compound (72 mg). The isolated compound was subjected to NMR and MS characterization. The molecular formula was assigned as C_5_H_5_NO_2_ by interpretation of a deprotonated molecular ion peak at *m*/*z* 110.0233 [M–H]^−^ in the HR-MS spectrum. The NMR spectral data is shown in Supplementary Information, and the compound was identified to be pyrrole-2-carboxylic acid, which was further confirmed by comparing with the standard compound.

### 2.5. Chitin Supplementation to Bacterial Cultures

To mimic the fungal cell wall components, chitin powder (0–2% *w*/*v*) from shrimp shells (Sigma Aldrich) was added to cultures of *Lysobacter* sp. 3655 grown in 3 mL of Martin medium. After 24 h of fermentation at 230 rpm at 30 °C, the cultures were extracted with acidified butanol (0.05% TFA) and analyzed using HPLC according to conditions stated above.

### 2.6. Antimicrobial Assays

To test the antibacterial activity of P2C, *Bacillus subtilis* was incubated in LB medium (3 mL) at 37 °C, and a 1% aliquot of the culture was mixed with 20 mL LB agar medium, which was poured into a plate for solidification. A volume of 5 µL of varying P2C concentrations (0 to 90 mM) was added to sterilized small circular filter papers. Kanamycin (Thermo Fisher Scientific, Fair Lawn, NJ, USA) (100 µg/mL) was used as control. The filter papers were placed on the LB agar plates and were incubated at 37 °C until inhibition zones were observed at 24 h.

To test the antifungal activity of P2C, each of the fungal strains, *Fusarium graminearum* and *Fusarium verticillioides*, was inoculated to a PDA plate (20 mL) and allowed to grow for 2 days in the static incubator at 30 °C. On the third day, a volume of 5 µL of varying P2C concentrations (0 to 20 mM) was added to sterilized small circular filter papers. A HSAF extract from *Lysobacter enzymogenes* OH11 was used as control. The plates were incubated at 30 °C until inhibition zones were observed after 7 days.

### 2.7. Microtiter Plate Biofilm Formation Assay

The OD_600_ of the overnight bacterial culture of *Lysobacter* sp. 3655 grown in LB containing kanamycin was measured and diluted to obtain a stock culture with an OD_600_ of 0.5. This was further diluted 50 times into a fresh 10% TSB medium, to obtain the starting culture with an OD_600_ 0.01. Three independent experiments were conducted for the biofilm formation assays, which were carried out in polystyrene 96-well microtiter plates. The schematic layout of the assays is given in Table S2 where column 1 contained 155 µL of the 10% TSB medium (blank) and column 2 consisted of 155 µL of diluted bacterial inoculate (control). Columns 3 to 12 consisted of 150 µL of serial diluted bacteria and 5 µL of P2C with varying concentrations (from 0.09 to 0.90 mM). The plates were covered with a low evaporation lid and were incubated at 30 °C under static conditions for 72 h.

After incubation, the OD_600_ was measured using the Synergy H1 Microplate reader (BioTek Instruments Inc., Winooski, VT, USA). Then, the cultures were pipetted out carefully to not disturb the biofilms. The plates were then washed twice with distilled water by immersing in a tub of water to remove any media and unattached bacterial cells in the wells. The wells were then stained with 165 µL of 0.1% crystal violet (CV) solution and incubated at room temperature for 30 min. The plates were rinsed with water three times and were turned upside down on paper towels and kept drying overnight. To quantify the biofilms, a portion of 165 µL of 33% acetic acid was added to each well to solubilize the crystal violet stains. The plates were incubated at room temperature for 30 min. The absorbance of dissolved CV was measured at 590 nm using the Synergy H1 Microplate reader.

The degree of biofilm production was calculated using Specific Biofilm Formation (SBF) equation given below, following the previously reported method [26]. There are 3 categories of the biofilm formation: (1) SBF ≤ 0.5, weak; (2) 0.5 > SBF ≤ 1, moderate; (3) SBF > 1, strong.

$$\begin{array}{c} Specific\;Biofilm\;Formation\\ =\frac{{OD}_{590}\;of\;attached\;and\;stained\;bacteria-{OD}_{590}\;of\;stained\;control\;well\;with\;no\;bacteria}{{OD}_{600}\;of\;bacterial\;cell\;growth\;in\;media} \end{array}$$

### 2.8. Microscopic Images of Biofilm

To observe the impact of P2C on biofilm formation, microscopic images of bacteria with and without P2C were obtained. As a comparison, the OH11 wildtype strain and the Δclp strain were included in the experiments, along with 3655 wildtype strain. The mutant strain Δclp is known to produce an excessive amount of adhesive biofilm [27]. The bacteria were cultured overnight in LB containing kanamycin, and an aliquot was transferred to 2 mL of 10% TSB medium to obtain an initial OD_600_ of 0.05. The experiment was carried out in 12-well polystyrene cell culture plates with a sterilized 11 mm × 11 mm plastic coverslip inserted for biofilm formation imaging. The cultures were kept in a static incubator for 72 h at 30 °C closed with a low evaporation lid. After incubation, the cultures were pipetted out, and the plates along with the coverslips were washed with water and stained with 0.1% CV and incubated at room temperature for 30 min. The coverslips were rinsed with water and imaged at 10× magnification using the Invitrogen EVOS M7000 (Bothell, WA, USA) epifluorescence microscope using the colored camera.

### 2.9. Statistical Analysis

All experiments were performed in duplicate or triplicate. Statistical analysis of the data was performed using GraphPad Prism (v9) software. A one-way ANOVA was used to determine the statistical significance between two data sets with one variable. A *p*-value less than 0.05 was considered as significant.

## 3. Results and Discussion

### 3.1. Co-Culture of Lysobacter and Fungus Led to Drastic Change of a Lysobacter Metabolite

**Figure 1:**
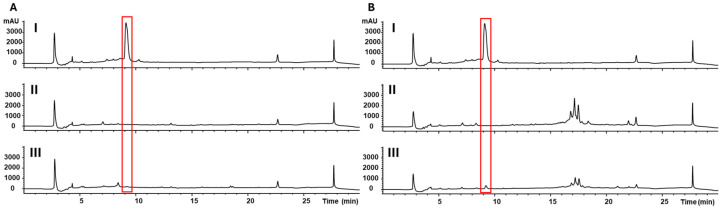
HPLC analysis of metabolites extracted from *Lysobacter* alone (**I**), fungus alone (**II**), or co-culture (**III**). (**A**). *Lysobacter–Fusarium graminearum* co-culture. (**B**). *Lysobacter–Fusarium verticillioides* co-culture. The red box highlights the *Lysobacter* metabolite that is suppressed by the fungus in co-culture.

To understand interactions between the environmental bacterial *Lysobacter* and fungal species, we devised a dialysis-based co-culture setup. This simple setup is convenient for studying small molecule signaling between microorganisms, as it avoids physical contact but allows molecules within certain sizes to move between microorganisms. *Lysobacter* was grown in the dialysis bag with a molecule weight cut-off (MWCO) of 6000–8000 Da, whereas the fungus was maintained in a culture flask. As controls, single microorganism cultures were also grown in parallel. Metabolites were extracted from the co-cultures, as well as the single-microorganism cultures, and analyzed by HPLC (Figure 1). The single-*Lysobacter* cultures consistently produced a prominent peak at 9.1 min on HPLC, which was not detectable in the single-fungus cultures. This predominant peak disappeared from the co-cultures, regardless of the fungal species in the co-cultures, *F. graminearum* or *F. verticillioides*. This suggests that the abolishment of this major metabolite in *Lysobacter* is due to the presence of fungi in the environment.

### 3.2. Structural Determination of the Lysobacter Metabolite

**Figure 2:**
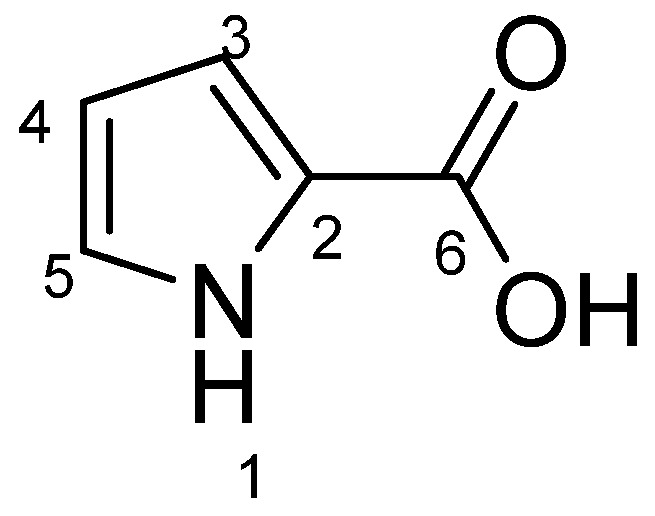
Structure of pyrrole-2-carboxylic acid (P2C).

This drastically changed *Lysobacter* metabolite, upon co-culturing with fungi, is very intriguing. We subsequently carried out scale-up cultures of *Lysobacter* sp. 3655 and prepared a pure sample (72 mg from 2 L Martin medium) through a series of column chromatography. The isolated compound was subjected to spectroscopic analyses. High resolution mass spectrometry (HR-MS) showed a molecular ion at *m*/*z* 110.0233 [M–H]^−^, with the molecular formula of C_5_H_5_NO_2_ (cal. 111.0320) (Figure S1). The spectral data from ^1^H-NMR, ^13^C-NMR, ^1^H-^1^H COSY, HSQC, and HMBC determined the compound to be pyrrole-2-carboxylic acid (P2C) (Figure 2 and Figures S2–S6, Table S2).

P2C is small enough to freely pass through the dialysis membrane and into the fungal culture in the flask. To test if the disappearance of P2C in *Lysobacter*, which was kept in the dialysis bag, was due to a diffusion of the compound into the flask, we checked the extracts from the fungal culture in the flasks after the co-cultures. The data showed that the extracts did not contain the P2C peak. This result suggests that the disappearance of P2C is likely due to a specific change in the metabolism of *Lysobacter* upon interacting with certain fungal molecules that could pass through the dialysis membranes.

### 3.3. Components of Fungal Cell Walls Contributed to P2C Suppression in Lysobacter

To find clues about what fungal factors that might have contributed to the P2C abolishment in *Lysobacter*, we tested chitin and *N*-acetyl glucosamine (GlcNAc). The fact that two different fungal species exhibited the same effect on P2C in *Lysobacter* culture implies that factors common to these fungi might be involved in this process. Chitin is a major component common to fungal cell walls, and GlcNAc is the monomer of chitin. We previously observed that *Lysobacter* is highly responsive to the presence of chitin and GlcNAc in the environment [16].

**Figure 3:**
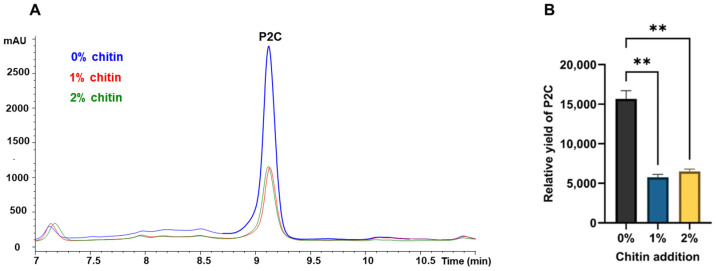
Suppression of P2C production in *Lysobacter* by chitin. (**A**). HPLC analysis of metabolites extracted from *Lysobacter* cultures supplemented with different concentrations of chitin. The blue trace represents the bacterial culture with 0% chitin, the red trace with 1% chitin and green trace with 2% chitin. (**B**). Quantification of the relative yield of P2C in the cultures. The relative yield was calculated based on the HPLC peak area of P2C divided by the OD_600_ value of the culture. For statistical data, ** *p* < 0.01.

When chitin was added to the single-*Lysobacter* culture, the P2C production was significantly reduced (Figure 3). However, P2C production was not totally abolished in the presence of chitin, suggesting that chitin is not the sole factor in the fungi responsible for the P2C repression in *Lysobacter* and there are other fungal factors contributing to this phenomenon. When the monomer GlcNAc was added to the single-*Lysobacter* culture, the P2C production was also reduced (Figure S7). But, the reduction was at a lower level than when chitin was added.

### 3.4. Antimicrobial Activity Assays for P2C

To understand the role of P2C in *Lysobacter*–fungus interactions, we conducted antibiotic assays. The antifungal assay was carried out in PDA medium using the same fungal strains used in the co-culture study to verify if the fungal growth would be impacted by P2C. No inhibition was observed in either fungal strain even when the P2C concentration reached 20 mM (Figure S8A). The antibacterial assay was conducted using *Bacillus subtilis* in LB medium. No inhibition zones were observed even at a concentration of 90 mM (Figure S8B). P2C was reported to cause growth inhibition of various bacterial strains including *Listeria monocytogenes*, with a MIC value of 6.75 mM [28]. The result suggests that P2C might exhibit antibacterial activity only to certain strains of bacteria.

### 3.5. Role of P2C in Biofilm Formation

To find clues for P2C’s potential function, we assessed the impact of P2C on the growth of *Lysobacter* itself. Standard P2C was exogenously added to the cultures, and their growth was measured after 24 h in two different media (Figure S9). Martin medium was used since the initial co-culture study was carried out in Martin medium, and 10% TSB medium was used as it is commonly used in *Lysobacter* research. The main difference between the two media is the nutrient concentration (mainly glucose), where Martin medium contains more glucose than 10% TSB medium. This impacted on the bacterial growth as well as the endogenous production of P2C. *Lysobacter* produced more P2C in Martin medium (4.32 ± 0.54 mM) than in 10% TSB (0.69 ± 0.054 mM). Correspondingly, *Lysobacter* strain 3655 could withstand higher exogenous P2C concentrations when cultured in 10% TSB medium (approximately 0.81–0.90 mM) compared to that in Martin medium (approximately 0.54–0.63 mM). When exogenous P2C was higher than the respective concentrations, the growth of *Lysobacter* strain 3655 in the media was completely inhibited (Figure S9).

**Figure 4:**
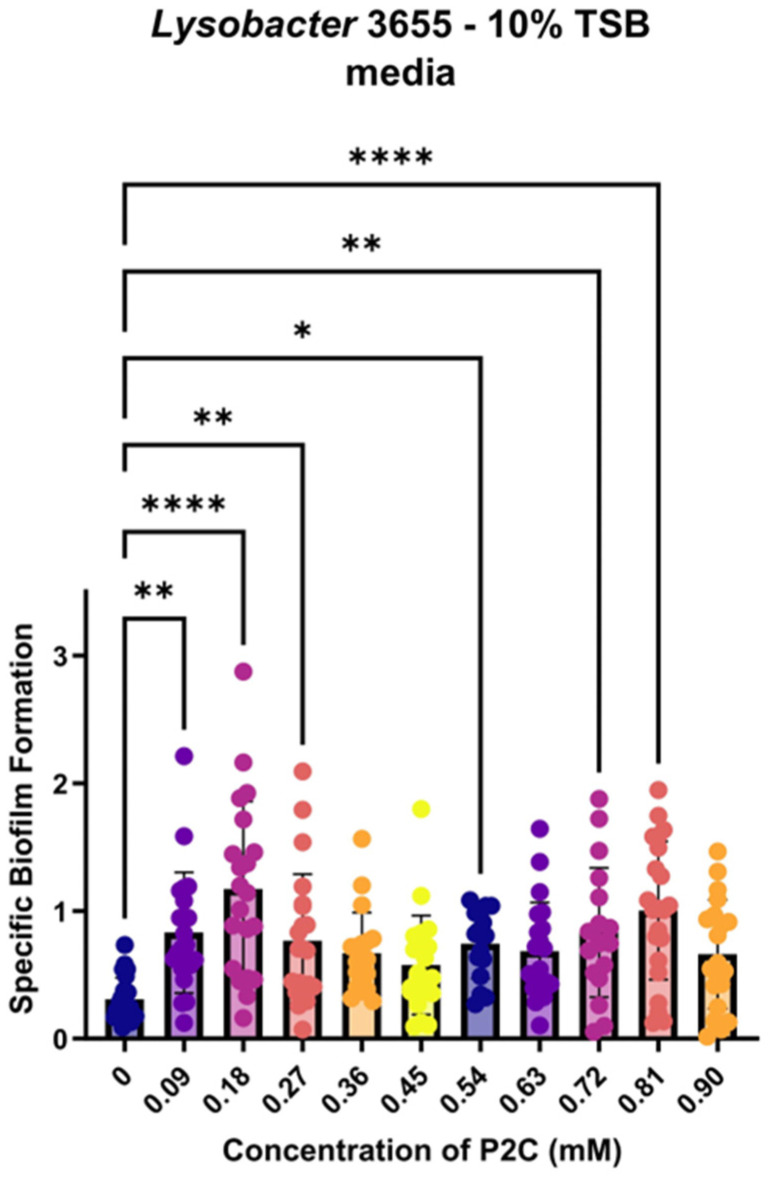
Effect of P2C on specific biofilm formation (SBF) of *Lysobacter* sp. 3655 cultures in 10% TSB. Each dot is one data point for that experimental condition. Each experimental condition consisted of 8 replicates, and the experiment was replicated 3 times yielded 24 data points for each experimental condition (with one condition shown in one color). For statistical data, * *p* < 0.05, ** *p* < 0.01, **** *p* < 0.0001.

As *Lysobacter* abolished or significantly reduced P2C production in the presence of fungi or chitin, we figured P2C might serve as a signal for *Lysobacter* to switch the growth mode during bacteria–fungal interactions. A phenomenon linked to bacterial growth is the formation of biofilms. It is where microbial communities adhere to surfaces with the production of extracellular polymeric substances. Thus, we assessed the effect of P2C on biofilm formation of *Lysobacter* strain 3655 (Figure 4). Quantitative analysis of *Lysobacter* biofilms is challenging due to the highly sensitive nature of the biofilm formation process in which numerous unknown factors could affect biofilms. This is evident from the relatively large deviations in the replicates of the experiments. Thus, we adopted specific biofilm formation (SBF) [26] to assess the effect of different P2C concentrations. As shown in Figure 4, P2C exhibited a concentration-dependent effect on SBF of *Lysobacter* strain 3655. When the concentration was below 0.18 mM, P2C significantly increased the SBF; when P2C concentration was higher than 0.18 mM, the SBF values started to drop, although the overall levels were still higher than the control. The data suggests that P2C might participate in signaling between free-cell growth and biofilm formation of *Lysobacter*, since small molecule signals often exhibit concentration-dependent effects [29]. It should be noted that the gradual increase of the SBF at high P2C concentrations (0.63–0.81 mM) might reflect the inhibitory activity of P2C at higher concentrations against *Lysobacter*. The inhibition led to a lower cell density (OD_600_), which inversely resulted in a higher calculated SBF based on the formula of SBF.

**Figure 5:**
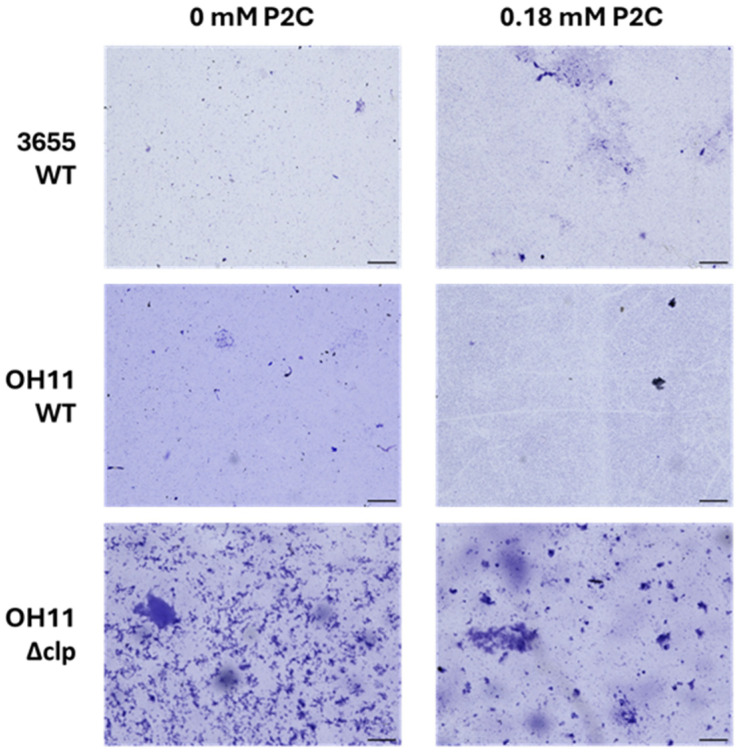
Effect of P2C on *Lysobacter* adhesion to plastic coverslips stained with crystal violet. Three strains were used, *Lysobacter* sp. 3655 wildtype, *L. enzymogenes* OH11 wildtype, and OH11 *clp* deletion mutant Δclp. Representative epifluorescence microscopic images were taken under color mode with 10× magnification. Scale bar = 100 µm.

To further understand the role of P2C, we conducted biofilm formation assays for *Lysobacter enzymogenes*, in which the global regulator Clp is known to be involved in biofilm formation [30]. Clp is a cyclic adenosine monophosphate (cAMP)-receptor-like protein. Previous studies showed that the *clp* gene controls the biofilm formation, in addition to in many other functions such as gliding motility, production of antifungal compounds, and lytic enzymes [27]. The *clp* deletion strain, Δclp, was reported to remarkably increase the cell adhesion to surfaces. We utilized the plastic coverslip method to study surface adhesion of three strains, *Lysobacter* sp. 3655, *L. enzymogenes* wildtype, and Δclp in the absence or presence of P2C. The results from epifluorescence microscopic images showed that P2C increased the cell density of the wildtype of both *Lysobacter* sp. 3655 and *L. enzymogenes* on the coverslip stained with crystal violet (CV) (Figure 5). This qualitative result is consistent with the quantitative results obtained from the SBF assays (Figure 4). As expected, the Δclp mutant produced significantly denser CV stains on the coverslips than the wildtype strain. This agrees with the previously reported data [27]. However, the P2C treatment of the Δclp mutant resulted in a clear reduction of CV staining, which implies that there might be competing pathways in *Lysobacter* strains that control the biofilm formation. Further studies are needed to get a deeper understanding of how the small molecule P2C interacts with the global regulatory protein during the regulation of biofilm formation.

Microbial co-culture is a valuable technique for investigating interactions between bacteria and fungi. By simulating microbial interactions, co-culturing enables the study of dynamic interspecies relationships while also facilitating the discovery of novel natural products, signaling pathways, and regulatory networks [31]. These microbial interactions can range from competitive to mutualistic, leading to significant alterations in the physiology and behavior of the organisms involved [32]. Previous research from our group explored predator–prey interactions between *Lysobacter enzymogenes* OH11 and fungal pathogens [16]. We found that the outer membrane vesicles (OMVs) produced by OH11 could mediate the antifungal activity. The OMVs serve as delivery vehicles for antifungal compounds such as HSAF, as well as chitin lytic enzymes. Chitin is a polymer of *N*-acetylglucosamine (GlcNAc) and a main structural component of fungal cell walls. Its degradation not only effectively disrupts fungal cell wall integrity but also releases oligomers of chitin that serve as inducers for more HSAF production. This results in more fungal inhibition by HSAF and more chitin degradation by the lytic enzymes that are co-delivered with HSAF in the OMVs.

In this study, we evaluated the bacterial–fungal interaction (BFI) between *Lysobacter* sp. 3655 and two fungal species, *Fusarium graminearum* and *F. verticillioides*, which are important in agriculture and economy. We designed a new set of experiments by using a non-HSAF producing strain, *Lysobacter* sp. 3655, in a dialysis tubing co-culturing with a fungal species in a flask. This setup not only prevents direct contact between *Lysobacter* and the fungus but also blocks OMVs and the exchange of macromolecules such as lytic enzymes. The system provides a unique opportunity to elucidate new strategies employed by *Lysobacter* species during BFI. Indeed, our work led to identification of pyrrole-2-carboxylic acid (P2C), which is produced in large quantities by *Lysobacter* sp. 3655. P2C has recently been reported in *L. enzymogenes* LE16 and *L. gummosus* YMF3.00690, with weak antagonistic effects on fungi and nematodes [33,34]. The most intriguing finding of this study is that the P2C production is drastically suppressed by the presence of fungi in the *Lysobacter* culture. Given that the two different strains of *Fusarium* fungi produce distinct mycotoxins—*F. graminearum* produces deoxynivalenol (DON) and analogs, while *F. verticillioides* produces fumonisins—it seems unlikely that the suppression of P2C was due to individual mycotoxins, as this phenomenon was observed with both fungal strains. This suggests that the suppression may be linked to a shared characteristic, such as a component of the fungal cell walls. Indeed, we found exogenously added chitin could significantly suppress the P2C production in *Lysobacter*. Since chitin from the fungi and lytic enzymes produced by *Lysobacter* are unlikely to cross the dialysis tubing, the results from co-culturing suggest that chitin-degrading enzymes of fungal origin might have contributed to the observed P2C suppression. It seems probable that small products of chitin degradation would cross the dialysis membranes and trigger the P2C suppression in *Lysobacter*. Additionally, there may be other mechanisms contributing to the P2C suppression, because chitin partly blocked P2C production while the fungal co-culture almost completely blocked the P2C production in *Lysobacter*.

P2C has been isolated from other bacteria including *Streptomyces griseus* and endophytic *Bacillus cereus* [35,36]. It was reported to exhibit broad-spectrum antimicrobial activity, with efficacy against multiple pathogenic microorganisms, including the oomycete plant pathogen *Phytophthora capsici* and the foodborne Gram-positive bacterium *Listeria monocytogenes* [28,35,37]. Previous studies suggested that the antimicrobial mechanism of P2C involves disruption of bacterial cell membranes, structural alterations of membrane-associated proteins, and perturbation of membrane lipid integrity as observed in *L. monocytogenes* where P2C reportedly had an MIC of 6.75 mM [28]. Additionally, P2C inhibited the growth of *P. capsici* by suppressing mycelial development and zoosporangia formation [35]. In this study, we found that, while *Fusarium graminearum* and *F. verticillioides* can dramatically suppress P2C, this molecule did not appear to inhibit the growth of the fungi, even at 20 mM. Moreover, P2C did not apparently inhibit the growth of *Bacillus subtilis* at 90 mM. Under the experimental conditions tested, *Lysobacter* sp. 3655 cultures produced P2C at 4.32 ± 0.54 mM in Martin medium and 0.69 ± 0.054 mM in 10% TSB medium. These concentrations are not sufficient to block the growth of the co-cultured Fusaria strains or *Bacillus subtilis*.

In *L. monocytogenes*, P2C was reported to function as a modulator of biofilm formation, through inhibiting the synthesis of extracellular polymeric substances (EPS) [38]. In *Pseudomonas aeruginosa*, P2C acted as a quorum sensing inhibitor (QSI) by reducing the production of virulence factors such as pyocyanin and proteases without compromising bacterial viability [39]. This quorum sensing interference extended to the disruption of gene expression associated with bacterial communication and pathogenesis. These findings underscore multi-facet properties of P2C. In this study, we found that P2C could control the growth of the producer, with inhibition observed at a concentration of 0.63 mM in Martin medium. Given the established link between growth regulation and biofilm formation, this suggests that P2C might influence biofilm development as well. Indeed, we found that P2C could significantly increase the SBF of *Lysobacter* sp. 3655. This effect was concentration dependent, with SBF continuing to increase when P2C concentration was below 0.18 mM and to decrease when concentration was over 0.18 mM. It is not uncommon that a signal molecule exhibits opposite effects at different concentration [29]. In addition, the addition of P2C led to a significant increase in biofilm formation in *L. ezymogenes*. However, in the global regulator mutant Δ*clp* of *L. ezymogenes*, P2C supplementation resulted in a clear reduction in biofilm formation, suggesting that P2C may function as a negative regulator in this genetic background. These findings highlight a potential regulatory role of P2C in modulating biofilm formation in *Lysobacter* species. Further research is needed to elucidate the interactions between the P2C-mediated biofilm stimulation and the Clp-mediated biofilm promotion.

## 4. Conclusions

*Lysobacter*, a genus of gliding bacteria within the soil microbiome, has attracted attention for its potential as a biocontrol agent due to its ability to produce a diverse array of bioactive natural products and extracellular lytic enzymes. These attributes position *Lysobacter* as a promising organism for the development of sustainable agricultural practices aimed at reducing the reliance on chemical pesticides. *Fusarium* wilt and root rot diseases affect a broad range of economically important crops, leading to extensive economic losses worldwide. In this study, we shed new light on the complex regulatory role of P2C in *Lysobacter* sp. 3655 and its interactions with *Fusarium* species. While several antifungal metabolites have been reported from *Lysobacter*, the role of pyrrole-containing compounds, particularly pyrrole-2-carboxylic acid (P2C), was not known in *Lysobatcer*. P2C controls bacterial growth and biofilm formation, highlighting its role in microbial interactions and adaptive mechanisms against fungal-derived environmental challenges.

## Data Availability

The original contributions presented in this study are included in the article/Supplementary Materials. Further inquiries can be directed to the corresponding author.

## Supplementary Materials

The following supporting information can be downloaded at: https://www.mdpi.com/article/10.3390/microorganisms13061202/s1, Table S1: Bacterial and fungal strains used in this study; Table S2: Layout of the 96-well plate for biofilm study; Figure S1: HR-MS of P2C isolated from *Lysobacter* sp. 3655; Table S3: ^1^H-NMR and ^13^C-NMR Spectroscopic Data for P2C in DMSO-*d*6 (500/125 MHz); Figure S2: ^1^H-NMR of P2C isolated from *Lysobacter* sp. 3655; Figure S3: ^13^C-NMR of P2C isolated from *Lysobacter* sp. 3655; Figure S4: ^1^H-^1^H COSY of P2C isolated from *Lysobacter* sp. 3655; Figure S5: HSQC of P2C isolated from *Lysobacter* sp. 3655; Figure S6: HMBC of P2C isolated from *Lysobacter* sp. 3655; Figure S7: HPLC chromatograms showing the impact of *N*-acetyl glucosamine (GlcNAc) on P2C production in *Lysobacter* sp. 3655; Figure S8: Assays for P2C’s antimicrobial activity. (A) Antifungal assay for P2C using *F. graminearum* and *F. verticillioides*. The schematic shows the different concentrations of P2C added to the filter papers. The control was a crude extract containing Heat Stable Anti-Fungal Factor (HSAF). (B) Antibacterial assay for P2C using *B. subtilis*. Kanamycin (Kan) was used as a positive control, and various concentrations of P2C were added on filter paper; Figure S9: Effect of P2C on the growth of *Lysobacter* sp. 3655 in Martin medium (A) and 10% TSB medium (B). Standard P2C was exogenously added to the cultures to observe the changes of growth after 24 hours. The top two panels are images of the cultures in two different media containing various P2C concentrations, and the bottom panel shows the quantitative assays of the growth (OD600). Culture in Martin medium produces a larger amount (4.32 ± 0.54 mM) of P2C endogenously, while culture in 10% TSB medium produces a smaller amount (0.69 ± 0.054 mM) of P2C endogenously.
