# Supplementary material for: Identification of Pyrrole-2-Carboxylic Acid from the Biocontrol Agent Lysobacter Involved in Interactions with Fusarial Fungi
Source: Microorganisms. 2025 May 24;13(6):1202. doi: 10.3390/microorganisms13061202 (PMC12194822; doi:10.3390/microorganisms13061202)
Supplement: Supplementary file 1 [file microorganisms-13-01202-s001.zip › microorganisms-3576072-supplementary.pdf]

Supporting Information

Identification of Pyrrole-2-carboxylic Acid from the Biocontrol Agent *Lysobacter* Involved in Interactions with Fusarial Fungi

Vishakha Jayasekera [1,2], Yong Han [3,4] and Liangcheng Du [1,2,*]

[1]Department of Chemistry, University of Nebraska-Lincoln, Lincoln, NE 68588, USA; udesilvajayasekera2@huskers.unl.edu (V.J.); ldu3@unl.edu (L.D.)

[2]Nebraska Center for Integrated Biomolecular Communication, University of Nebraska-Lincoln, Lincoln, Nebraska, 68588, USA

[3]Edison Biotechnology Institute, Ohio University, Athens, OH 45701, USA; hany@ohio.edu (Y.H.)

[4]Department of Chemistry and Biochemistry, Ohio University, Athens, OH 45701, USA

*Correspondence: ldu3@unl.edu

Table S1: Bacterial and fungal strains used in this study.

Strains	Description	Source/Reference
**Bacteria**		
Lysobacter sp. 3655	Wild-type, Km^r	DSM
L. enzymogenes		
OH11	Wild-type, Km^r	CGMCC No. 1978
OH11 Δclp	*clp* global regulator gene deletion mutant	From the Yuen lab [1]
Bacillus subtilis	Strain used for antibacterial assay	Collection of the Du lab [2]
**Fungus**		
Fusarium graminearum	Wild type	From the Yuen lab [3]
Fusarium verticillioides	Wild type	Collection of the Du lab [4]

Table S2: Layout of the 96-well plate for biofilm study.

	**1**	**2**	**3**	**4**	**5**	**6**	**7**	**8**	**9**	**10**	**11**	**12**
**A**	Blank – 10% TSB medium only	Control – 3655 bacteria only	Test condition – 0.09 mM P2C	Test condition – 0.18 mM P2C	Test condition – 0.27 mM P2C	Test condition – 0.36 mM P2C	Test condition – 0.45 mM P2C	Test condition – 0.54 mM P2C	Test condition – 0.63 mM P2C	Test condition – 0.72 mM P2C	Test condition – 0.81 mM P2C	Test condition – 0.90 mM P2C
**B**												
**C**												
**D**												
**E**												
**F**												
**G**												
**H**												

Figure S1: HR-MS of P2C isolated from *Lysobacter* sp. 3655.

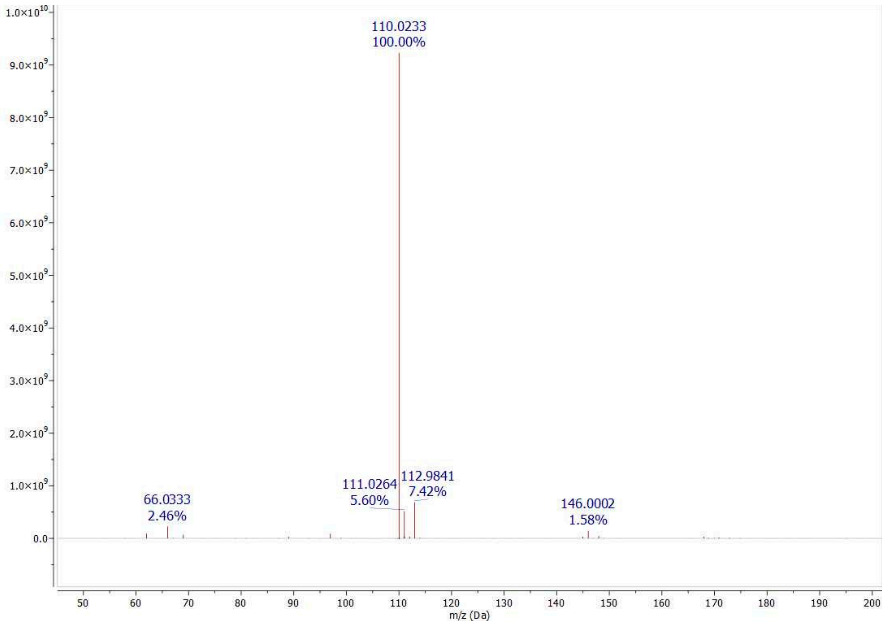

Table S3: ^{1}H-NMR and ^{13}C-NMR Spectroscopic Data for P2C in DMSO-d_6 (500/125 MHz)

**Position**	δ_H (***J* in Hz**)	δ_C**, type**
1-NH	11.70, s	
2		123.4, C
3	6.72 – 6.73	115.1, CH
4	6.13, d (2.5)	109.7, CH
5	6.95 – 6.96	123.9, CH
6		162.3, C
6-OH	12.40, s	

Figure S2: ^{1}H-NMR of P2C isolated from *Lysobacter* sp. 3655.

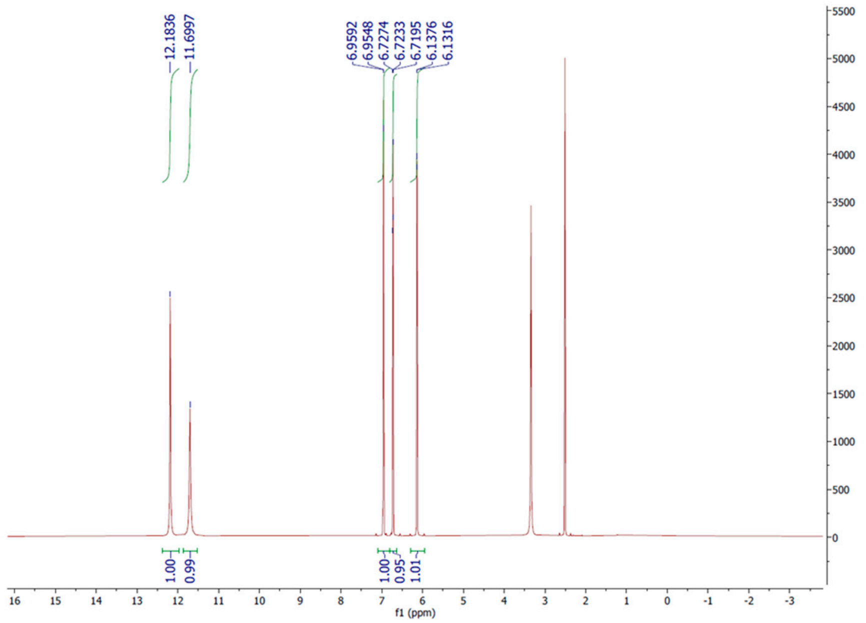

Figure S3: ^{13}C-NMR of P2C isolated from *Lysobacter* sp. 3655.

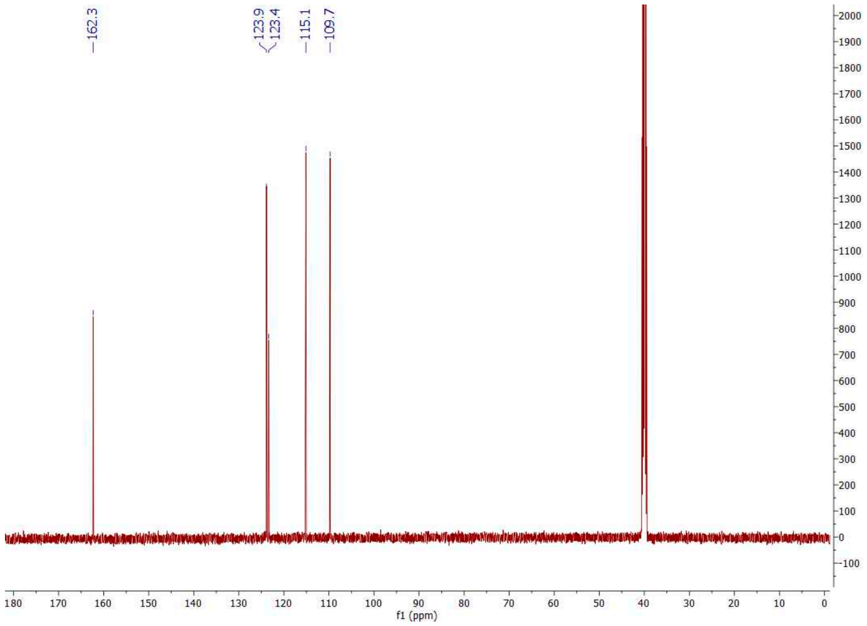

Figure S4: ^{1}H-^{1}H COSY of P2C isolated from *Lysobacter* sp. 3655.

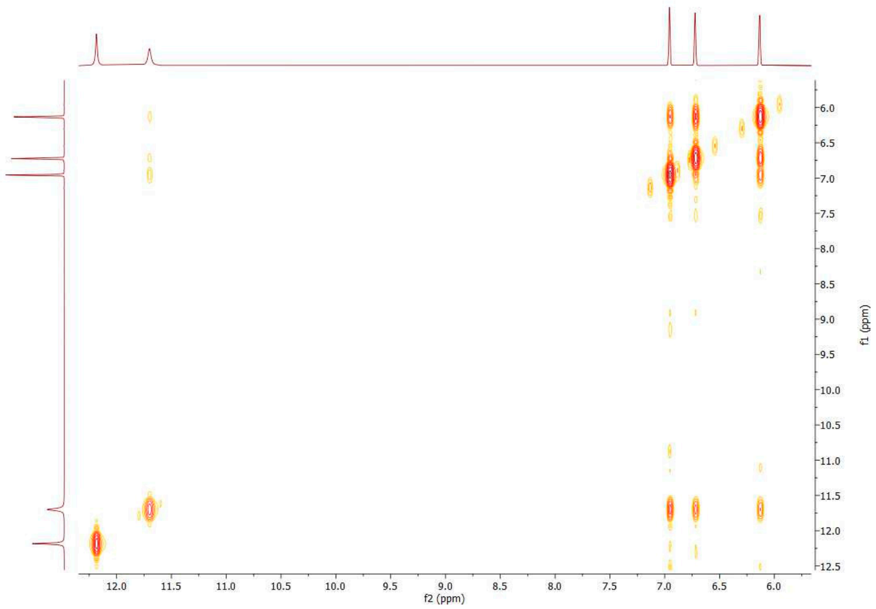

Figure S5: HSQC of P2C isolated from *Lysobacter* sp. 3655.

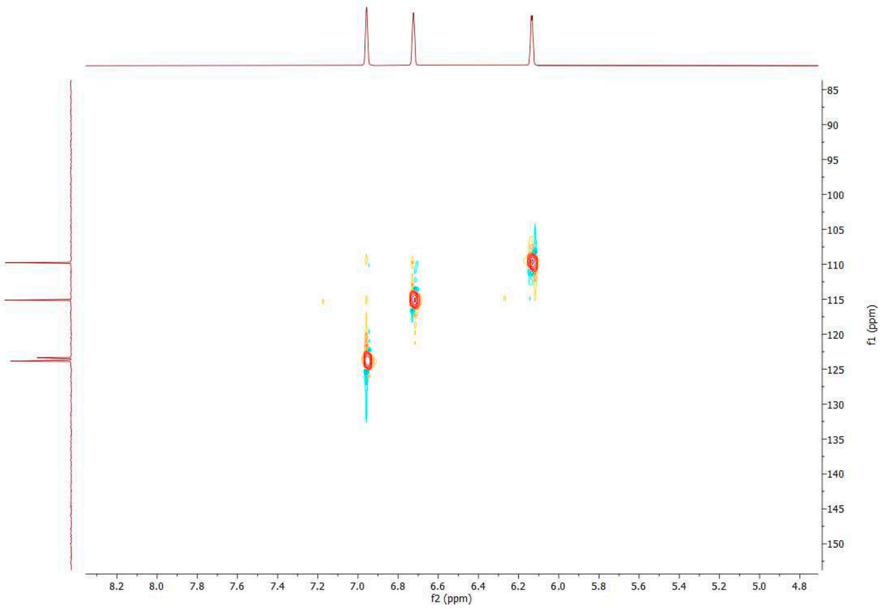

Figure S6: HMBC of P2C isolated from *Lysobacter* sp. 3655.

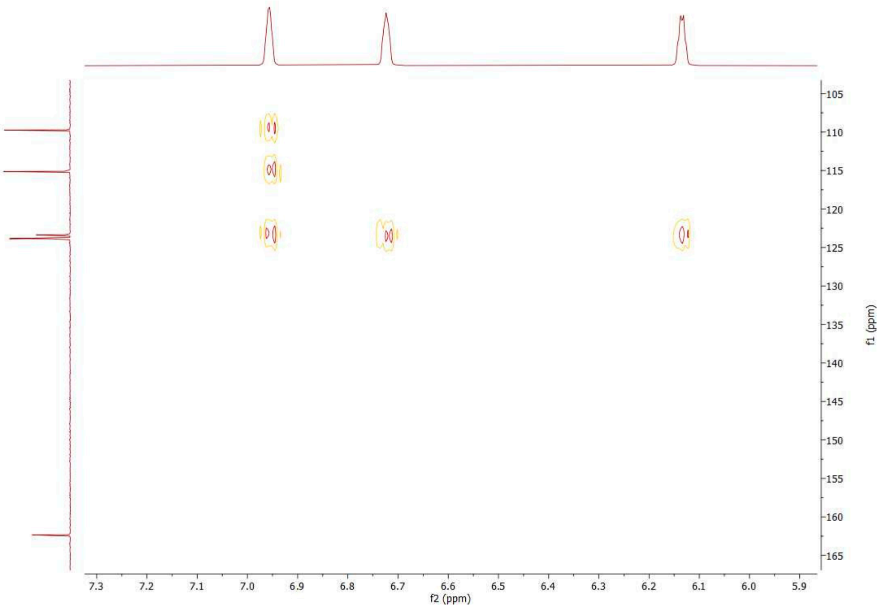

Figure S7: HPLC chromatograms showing the impact of *N*-acetyl glucosamine (GlcNAc) on P2C production in *Lysobacter* sp. 3655.

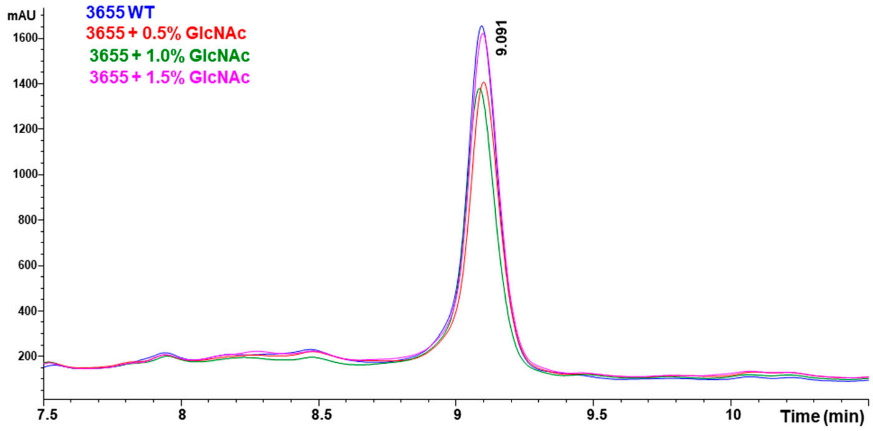

Figure S8: Assays for P2C's antimicrobial activity. (A) Antifungal assay for P2C using *F. graminearum* and *F. verticillioides*. The schematic shows the different concentrations of P2C added to the filter papers. The control was a crude extract containing Heat Stable Anti-Fungal Factor (HSAF). (B) Antibacterial assay for P2C using *B. subtilis*. Kanamycin (Kan) was used as a positive control, and various concentrations of P2C were added on filter paper.

(A)

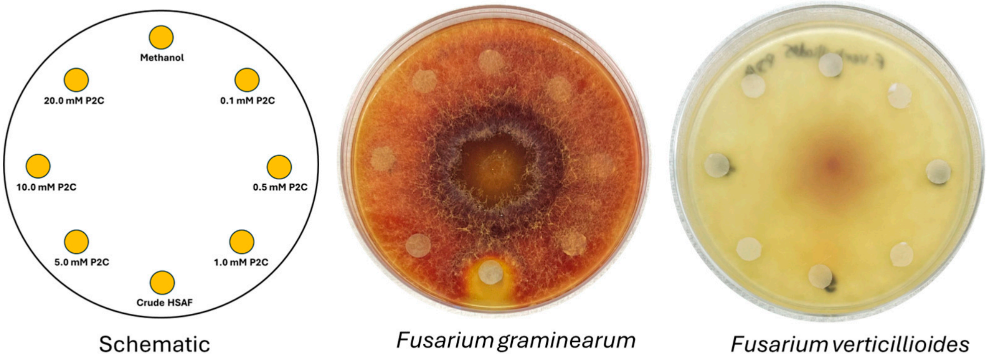

(B)

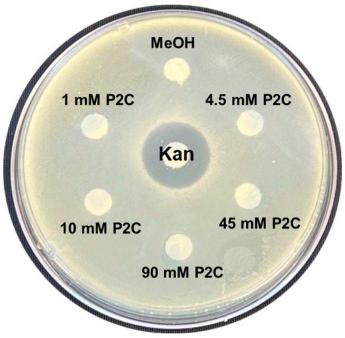

Figure S9: Effect of P2C on the growth of *Lysobacter* sp. 3655 in Martin medium (A) and 10% TSB medium (B). Standard P2C was exogenously added to the cultures to observe the changes of growth after 24 hours. The top two panels are images of the cultures in two different media containing various P2C concentrations, and the bottom panel shows the quantitative assays of the growth (OD_{600}). Culture in Martin medium produces a larger amount (4.32 ± 0.54 mM) of P2C endogenously, while culture in 10% TSB medium produces a smaller amount (0.69 ± 0.054 mM) of P2C endogenously.

(A)

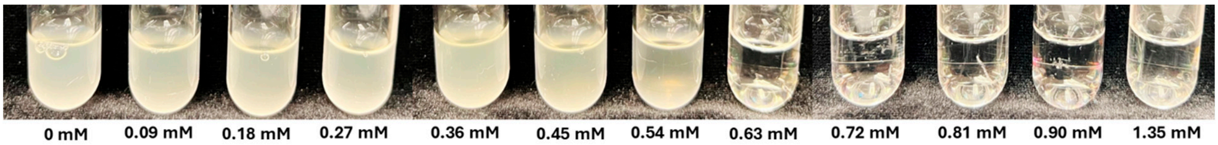

(B)

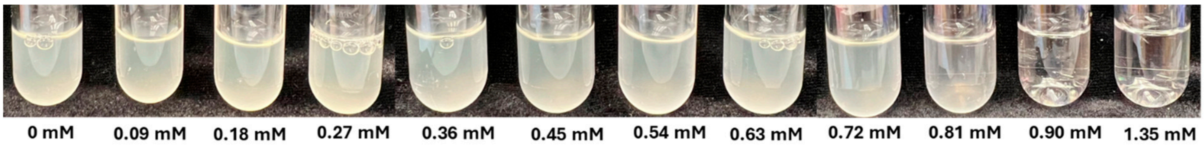

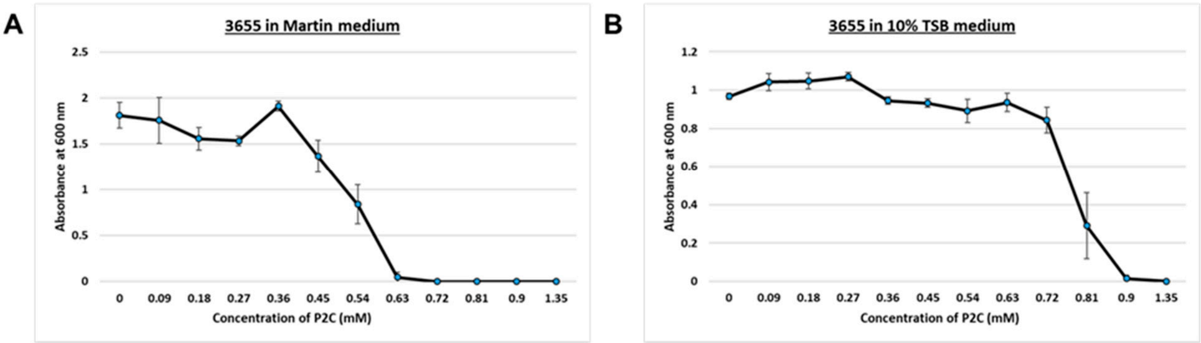

1. Kobayashi, D.Y.; Reedy, R.M.; Palumbo, J.D.; Zhou, J.M.; Yuen, G.Y. A *clp* gene homologue belonging to the *Crp* gene family globally regulates lytic enzyme production, antimicrobial activity, and biological control activity expressed by *Lysobacter enzymogenes* strain C3. *Appl Environ Microb* **2005**, *71*, 261-269.

2. Chen, X.S.; Li, S.R.; Yu, L.J.; Miller, A.; Du, L. Systematic optimization for production of the anti-MRSA antibiotics WAP-8294A in an engineered strain of *Lysobacter enzymogenes*. *Microb. Biotechnol.* **2019**, *12*, 1430–1440. https://doi.org/10.1111/1751-7915.13484.

3. Li, S.; Jochum, C.C.; Yu, F.; Zaleta-Rivera, K.; Du, L.; Harris, S.D.; Yuen, G.Y. An antibiotic complex from *Lysobacter enzymogenes* strain C3: antimicrobial activity and role in plant disease control. *Phytopathology* **2008**, *98*, 695-701.

4. Yi, H.; Bojja, R.S.; Fu, J.; Du, L. Direct evidence for the function of *FUM13* in 3-ketoreduction of mycotoxin fumonisins in *Fusarium verticillioides*. *J Agric Food Chem* **2005**, *53*, 5456-5460.
